# The effects of supplemented sericin on in vitro maturation and preimplantation development of mouse embryos: An experimental study

**DOI:** 10.18502/ijrm.v19i10.9824

**Published:** 2021-11-04

**Authors:** Omid Banafshi, Sherko Nasseri, Leila Farhadi, Masoud Alasvand, Mohammad Bagher Khadem-Erfan, Javad Hosseini, Saber Miraki, Fardin Fathi

**Affiliations:** ^1^Cellular and Molecular Research Center, Research Institute for Health Development, Kurdistan University of Medical Sciences, Sanandaj, Iran.; ^2^Cancer and Immunology Research Center, Research Institute for Health Development, Kurdistan University of Medical Sciences, Sanandaj, Iran.

**Keywords:** Sericin, In vitro maturation, In vitro fertilization, Preimplantation embryo, Culture medium, Mice.

## Abstract

**Background:**

Mouse embryo culture condition is an essential part of transgenic, reproductive and developmental biology laboratories. Mouse embryonic culture media may have a high risk of serum contamination with pathogens.

**Objective:**

To investigate the effect of sericin as an embryo culture medium supplement on in vitro maturation (IVM), in vitro fertilization (IVF), and development of the preimplantation embryo in mice.

**Materials and Methods:**

The effects of sericin at three concentrations (subgroups) of 0.1%, 0.5%, and 1% as a medium supplement on IVM, IVF, and in vitro development of mouse embryos were separately investigated and compared with a sericin-free (control) group. The cumulative effect of the three concentrations was evaluated for IVM + in vitro development and IVF + in vitro development as follow-up groups.

**Results:**

In the IVM group, compared to the control group, the number of oocysts reaching the MII stage was significantly higher when 1% sericin was used (161/208 = 77.4%). No significant results were observed in the IVF and in vitro development groups with different concentrations of sericin compared to the control group. Among the follow-up groups, in the IVM + in vitro development group, the number of oocytes was higher after passing the IVM and IVF and reaching the blastocysts stage when 1% sericin was used, compared with other sericin subgroups. A significant difference was also noted when compared with the control group (p = 0.048). The IVF + in vitro development study group, on the other hand, did not show any significant relationship.

**Conclusion:**

It can be concluded that 1% sericin can be used as a supplement in mouse embryo cultures to improve the IVM rate. Also, based on the findings, sericin appears to be an effective supplement which can have a positive effect on the development of embryos derived from IVM.

## 1. Introduction

In vitro culture of murine embryos has traditionally been a two-step process of retrieval and cultivation, requiring appropriate unique media for culture conditions. Embryos are routinely harvested from oviducts and placed into an embryo culture medium. In the next step, the embryos are transferred into a bicarbonate-based buffered medium placed in a culture incubator (1). Different types of primary culture media used for in vitro embryo culture are often provided with sera such as fetal bovine serum or bovine serum albumin for the preparation of growth factors, amino acids, hormones, and proteins which are useful for embryonic development (2). The high risk of sera contamination with pathogens, prions, viruses, and bovine spongiform encephalopathy, etc. has prompted researchers to try to find alternative supplements that can be used instead of serum during in vitro embryo culture (3, 4). Dash and colleagues found that sericin had an antioxidant effect on skin fibroblast cells exposed to hydrogen peroxide. They showed the potential to inhibit the production of intracellular hydrogen peroxide in ultraviolet-treated keratinocytes (5).

Sericin is a water-soluble silk protein extracted from cocoons and it is the second major protein component (besides fibroin) of silk used in biological materials due to its anti-UV and antibacterial properties. Sericin is composed of 18 types of amino acids as well as polar side groups such as carboxyl, hydroxyl, and amino groups and it is rich in serine and aspartic acid (6). Izobe et al. found that culturing two-cell embryos separately for seven days in an environment with 0.5% sericin resulted in the highest rate of blastocyst formation and development into expanded blastocysts. It has been shown that preimplantation development and the quality of cultured bovine embryos increased following the addition of sericin to an in vitro medium, which prevented oxidative stress (7). It was reported that supplementation of a medium with 0.1% sericin during in vitro maturation (IVM) improved the nuclear maturation and fertilizability of sheep oocytes. Also, because of its antibacterial and UV-resistant properties, as a biomaterial, it may replace bovine serum albumin in chemical environments without risk of disease transmission. Several studies have suggested the benefits of using sericin as a protein replacement (8). Also, some studies have suggested that sericin can have a role in cryoprotection in the freezing of mice, human, and buffalo sperm (9-11).

The present study is the first to investigate the effect of 0.1%, 0.5%, and 1% sericin as a medium supplement on IVM, the rate of in vitro fertilization (IVF), and in vitro development of mouse embryos.

## 2. Materials and Methods

### Sericin preparation and study groups

Sericin and the other chemicals used in this study were purchased from Sigma-Aldrich (St. Louis, MO, USA), and the culture-related plastics were purchased from Falcon (Paignton, UK). Sericin was diluted in a human tubal fluid medium (HTF). Sericin was considered as a supplement in the embryo culture medium at 0.1%, 0.5% and 1% concentrations (subgroups I, II, and III, respectively) in comparison with a sericin-free group as a control group. Four study subgroups were created within each experimental group: IVM, IVF, IVF + in vitro development, and IVM + in vitro development.

### Animals

32 NMRI mice were used to extract germinal vesicle (GV) oocytes, metaphase II (MII) oocytes, and sperm. Then, IVM, IVF, and evaluation of mouse embryo development in vitro were performed. The mice had free access to water and food and were preserved at a temperature of 23 ± 2°C and a 12-hr light/dark cycle. All processes were carried out at the Kurdistan University of Medical Science in 2018.

### Collection of immature GV oocytes and IVM GV oocytes and IVM

A total of 5 IU of pregnant mare serum gonadotropin (PMSG) was injected into each 4-6-wk-old female mouse. Then, 48 hr post injection, immature GV oocytes were released from the ovaries by puncturing the follicles as visualized under a stereomicroscope. The GV oocytes were collected and randomly classified into four subgroups: 0% (control) and 0.1%, 0.5%, and 1% HTF containing sericin (4). The GV oocytes were incubated at 37°C in a humidified atmosphere of 5% CO ${}_{2}$ in the air for 24 hr. Next, the oocytes displaying the first polar bodies by a stereomicroscope were considered as MII oocytes. The MII oocytes were then recruited for the IVM + in vitro development follow-up group (Table I).

### Collection of MII oocytes and IVF

IVF was performed as described previously with minor modifications (12). PMSG was injected into female mice that were 4-6 wk old. After 48 hr, human Chorionic Gonadotropin (hCG) was injected, and oocytes were collected from mouse oviducts 14 hr post the hCG injection. The mature oocytes (metaphase meiosis II) were transferred to 100-µl IVF medium droplets, and 1 × 10 ${}^{6}$ sperms/ml was added to the IVF droplets supplemented with sericin: 0% (control) and 0.1%, 0.5%, and 1% as test subgroups for 24 hr. Then, the zygotes were monitored by a reverse microscope and the percentage of two-cell formation was recorded to assess the rate of fertilization (Table I).

### Collection of zygotes and in vitro development

After the PMSG and hCG injections, the injected female mice mated with male mice. 18 hr post hCG injection, female mice with a vaginal plaque were selected and euthanized with cervical dislocation. The two-pronucleus-stage embryos were collected from the mouse oviducts, observed, and screened at × 100 magnification on a warmed microscope stage (37°C); they were not fragmented or degenerated and were classified into three subgroups of 0.1%, 0.5%, and 1% sericin added potassium-supplemented simplex optimized medium (KSOM) and non-added sericin as a control group (with KSOM). Each plate contained 200 ul of KSOM covered by preincubated mineral oil used for two-cell-stage embryo culture and development at 37°C in 5% CO ${}_{2}$ in the air inside a humidified incubator (10). The number of zygotes developing to early blastocysts was calculated in each sericin subgroup.

### Follow-up from IVM to the blastocyst stage

For assessing the cumulative effect of sericin as an embryo culture supplement, we used the MII oocytes obtained from the IVM study group for IVF and subsequent embryo development. During all three stages of the experiment, the culture media used were supplemented with three different concentrations of sericin (13). The number of obtained blastocysts was considered as a complete process of oocyte maturation, fertilization, and in vitro embryo development in the presence of sericin. It was then compared to the control group.

### Follow-up from IVF to the blastocyst stage

To assess the cumulative effect of sericin as an embryo culture supplement in addition to IVF, the medium for the culture of the subsequent embryo from IVF was supplemented with three different concentrations of sericin. The number of blastocysts was considered as a complete process of IVF of MII oocytes and development to blastocyst in the presence of sericin and compared to the control group.

**Table 1:** Summary of materials and methods and experiments performed


**TEST**	**Sericin concentration**	**Culture medium used**	**Stage of cultured oocyte/embryo**	**Stage of obtained oocyte/embryo**
**IVM**		GV	MII
**IVF**		MII	Two cell
**In vitro development**	HTF	Two-pronucleus embryo	Blastocyst
**IVM to blastocyst**			GV	Blastocyst
**IVF to blastocyst**		0.1% 0.5% 1% 0% (control)	HTF&KSOM	MII	Blastocyst
This study was performed with four experimental groups. The effects of sericin at four concentrations (subgroups) of 0% (control) and 0.1%, 0.5%, and 1% as a medium supplement on IVM (In vitro maturation), IVF (In vitro fertilization), and in vitro development of mouse embryos were separately investigated, HTF: Human tubal fluid medium, KSOM: Potassium simplex optimization medium, GV: Germinal vesicle, MII: Metaphase II oocytes				

### Ethical considerations

All procedures were carried out based on the guidelines of the Ethics Committee of the Kurdistan University of Medical Science, Sanandaj, Iran (Code: IR.MUK.REC.1395/215). According to the University Ethics Committee, working with laboratory animals in this study did not violate any rules.

### Statistical analysis

In this experimental study, the IVM and IVF rates and the percentage of development to the blastocyst stage (in vitro development) in mouse oocytes and embryos were calculated for each sericin group and subgroup, and these values were then compared with those of the control group. One-way ANOVA was run to assess group differences. Data were analyzed by Stata 14 (Stata Corp. 2015. Stata Statistical Software: Release 14. College Station, TX: Stata Corp LP). The significance level in this study was set at p < 0.05.

## 3. Results

### The effect of sericin as an embryo culture supplement on IVM, IVF, and the in vitro development rate

Initially, 832 GV oocytes were classified into 208 GV oocytes for each sericin subgroup and control group. Among the three sericin subgroups, the effect of 1% sericin (161/208 = 77.4%) was significant in comparison with the control sericin-free group (143/208 = 68.7%). On the other hand, the 0.1 and 0.5% subgroups did not show any significant relationship.

For IVF, a total of 124 oocytes were recruited for each sericin subgroup of 0.1, 0.5, 1% sericin, and the sericin-free control group for testing the effect of sericin on IVF. Notably, the one-way ANOVA analysis of the three sericin subgroups showed no significant relationship in comparison with the control group.

Regarding the in vitro development rate, after collecting the 688 two-pronucleus-stage embryos from the oviducts and dividing them into three subgroups based on sericin concentration and a control group (without sericin), the number of zygotes that developed into early blastocysts was calculated. One-way ANOVA analyses revealed that the three concentrations of sericin as an embryo culture supplement had no significant effect on the in vitro development of mice zygotes (Table II).

### The effect of sericin as an embryo culture supplement on mice embryo culture from GV oocytes to the blastocyst stage (IVM + in vitro development)

In this study, to assess the cumulative effect of sericin as an embryo culture supplement, the effect of 0.1%, 0.5%, and 1% sericin on the development of MII oocytes from the IVM study group to two-cell embryos (after IVF), and finally to the blastocyst stage was evaluated. The one-way ANOVA results revealed a significant relationship between HTF in which 1% sericin was added as a supplement and the follow-up of GV oocytes to blastocysts. Importantly, 0.1% and 0.5% sericin did not have significant effects (Table II).

### The effect of sericin as an embryo culture supplement on IVF of MII oocytes to the blastocyst stage (IVF + in vitro development rate)

The cumulative effect of sericin as an embryo culture supplement on IVM and then on the development to blastocysts was examined in the IVF + in vitro development study group. It was found that the rate of in vitro-fertilized MII oocytes reaching the blastocyst stage was not significantly different in the 0.1%, 0.5%, or 1% sericin subgroups than in the control group (Table II). The results showed that in the IVM group, the number of oocytes that reached stage MII was significantly higher in the 1% sericin subgroup (161/208 = 77.4%) compared to the control group. No significant difference was observed between the IVF and in vitro development of embryo groups with different concentrations of sericin compared to the control group (p = 0.047). In the IVM + in vitro development of embryo group, the number of oocytes was higher after passing IVM and IVF and reaching the blastocyst stage when 1% sericin was used compared to the other sericin subgroups. There was also a significant difference compared to the control group (p = 0.048). However, in the IVF + in vitro development of the embryo group, no significant relationship was found between the different subgroups.

**Table 2:** Effects of sericin concentrations on IVM, IVF, and in vitro development of the embryo, and follow-up groups including IVM and IVF + in vitro development of the embryo


**Study groups**		**Group I** **sericin 0.1**	**Group II** **sericin 0.5**	**Group III sericin 1 **	**Group IV control (without sericin)**
**IVM**					
	**Mature MII **	144 (69)	147 (70)	161 (77.4)	143 (68.7)
	**Immature MI**	64	61	47	65
	**Total**	208	208	208	208
**p-value**		0.916	0.670	0.047	
**IVF**					
	**No. of two-cells stage**	89 (71)	96 (77)	93 (75)	87 (70)
	**Unfertilized oocyst**	35	28	31	37
	**Total**	124	124	124	124
**p-value**		0.780	0.194	0.393	
**In vitro development**					
	**No. of blastocysts **	135 (78)	140 (81)	137 (79)	132 (77)
	**Undeveloped to blastocyst**	37	32	35	40
	**Total**	172	172	172	172
**p-value**		0.698	0.289	0.514	
**IVM + in vitro development**					
	**No. of blastocysts **	31 (21.5)	41 (28)	54 (33.5)	32 (22)
	**Undeveloped to blastocyst**	113	106	104	111
	**Total**	144	147	161	143
**p-value**		0.862	0.279	0.048	
**IVF + in vitro development**					
	**No. of blastocysts **	57 (71.3)	59 (73.8)	55 (68.8)	53 (66.3)
	**Undeveloped to blastocyst**	23	21	25	27
	**Total**	80	80	80	80
**p-value**		0.428	0.251	0.654	
Data are presented as n (%), One-way ANOVA, P < 0.05					

## 4. Discussion

In the present study, the effect of sericin on IVM, IVF, and in vitro development was determined separately. Further, its cumulative effect from immature oocytes to blastocyst development was assessed.

Our findings show that, while the IVM rate was higher in the group of supplementation with 1% sericin in comparison with the sericin-free group, adding 0.1% or 0.5% sericin to the medium did not show any significant effect on the maturation rate. The current study is the first to report the effects of sericin supplementation on mice oocyte maturation in culture. In line with our study, Do and colleagues showed that the addition of IVM medium with 1% sericin may improve the meiotic capacity of pig oocytes and the quality of blastocysts, which is determined by the DNA fragmentation index (14). Inconsistent with our results, a study showed that the addition of 0.5% sericin to an in vitro culture medium improved the full cumulus expansion rate and the percentage of oocytes that reached the metaphase-II stage in Sanjabi ewes. Also, they reported that supplementation of 0.1% sericin in the maturation culture had a significant effect on nuclear and cytoplasmic maturation, thereby increasing preimplantation development of in vitro-cultured embryos (10). Another study reported that using 0.5% sericin as an alternative protein supplement enabled the feasibility of IVM of bovine oocytes to be demonstrated (13).

In the current study, sericin was associated with a higher total formation rate of blastocysts from MII-stage embryos. It has been reported that sericin, by preventing oxidative stress during bovine embryo culture, helps to improve the embryo quality and increase embryonic development (15). The results of this study showed that adding 1% sericin to the culture medium had a significant positive effect on pre-implantation growth of embryos compared to other concentrations of sericin. Moreover, these results revealed that the percentages of MII oocyte maturation and development to blastocyst were lower at the low concentration (0.1%) of sericin compared to the control group. It seems that the sericin effect on the MII oocyte maturation and development to blastocyst is concentration-dependent. MII maturation and development to blastocyst in rats increased with higher sericin concentration. In another study, contrary to our results, the percentages of cleavage and blastocyst rates in ewe embryos were significantly lower at high concentrations (2.5%) of sericin treatments compared with the control group (16).

In another study, adding 0.5% sericin to the culture medium increased the blastocyst rate compared to the control group without sericin and acted as an alternative protein supplement for IVM of bovine oocytes and zygotes (4). In addition, some researchers have found that adding 0.5% sericin to an in vitro culture medium improves oxidative stress, pre-implantation progression, and the quality of embryos (7). In another study, it was found that supplementation with sericin during embryo culture in vitro enhanced the development rates of ovum pick-up-derived embryos cultured separately, and improved the quality of the bovine embryos specifically (15). However, our results showed that supplementation of the medium with different concentrations of sericin had no significant effect on the development of the embryo in vitro. The highest maturation and development rate was observed in the oocytes that matured in a medium supplemented with 1% sericin.

## 5. Conclusion

It can be concluded that the medium supplemented with 1% sericin improved the IVM and development of MII oocyte-derived embryos to blastocysts in the mouse model. To ensure optimal concentration, more investigations are needed.

##  Conflict of Interest 

The authors declare that there is no conflict of interest.
